# Geckos decouple fore- and hind limb kinematics in response to changes in incline

**DOI:** 10.1186/s12983-016-0144-2

**Published:** 2016-03-02

**Authors:** Aleksandra V. Birn-Jeffery, Timothy E. Higham

**Affiliations:** Department of Zoology, University of Cambridge, Downing Street, Cambridge, UK; Department of Biology, University of California, 900 University Avenue, Riverside, CA 92521 USA

**Keywords:** Gecko, Incline, Decline, Adhesive system, Forelimb, Hind limb

## Abstract

**Background:**

Terrestrial animals regularly move up and down surfaces in their natural habitat, and the impacts of moving uphill on locomotion are commonly examined. However, if an animal goes up, it must go down. Many morphological features enhance locomotion on inclined surfaces, including adhesive systems among geckos. Despite this, it is not known whether the employment of the adhesive system results in altered locomotor kinematics due to the stereotyped motions that are necessary to engage and disengage the system. Using a generalist pad-bearing gecko, *Chondrodactylus bibronii*, we determined whether changes in slope impact body and limb kinematics.

**Results:**

Despite the change in demand, geckos did not change speed on any incline. This constant speed was achieved by adjusting stride frequency, step length and swing time. Hind limb, but not forelimb, kinematics were altered on steep downhill conditions, thus resulting in significant de-coupling of the limbs.

**Conclusions:**

Unlike other animals on non-level conditions, the geckos in our study only minimally alter the movements of distal limb elements, which is likely due to the constraints associated with the need for rapid attachment and detachment of the adhesive system. This suggests that geckos may experience a trade-off between successful adhesion and the ability to respond dynamically to locomotor perturbations.

## Background

Animals move through complex environments, which require them to deal with a number of important factors, such as differing slopes, textures, and perturbations. However, most research focusing on locomotor biomechanics has examined level locomotion [1–6]. Because of this, it is unclear whether there is a common neuromechanical template for inclined locomotion across species, as has been observed for level locomotion [3, 7–9]. Although going up is critical for most terrestrial animals, coming back down may be just as critical, if not more important [10]. The locomotor strategies for descending are unknown, but may be enhanced by morphological features such as claws or adhesive structures [11].

Moving uphill requires an animal to move against gravity, requiring more work compared to level surfaces [12]. On inclines, a backwards toppling moment depends on the steepness of the slope, is caused by the rotation of the body with respect to gravity, along with the placement of the base of support, and must be overcome or reduced. The majority of species tend to adopt a more crouched posture to reduce this toppling moment on inclines, which is often achieved by greater flexion at the elbow and/or knee [13–16]. To account for the increased work requirement, and to reduce musculoskeletal stresses, the majority of animals also reduce their speed [14, 17–22], and increase duty factor [13, 23, 24] on inclines to increase contact time, mainly in order to decrease peak forces, but this may have consequences for improving the stability of the animal on inclines as well.

Unlike moving uphill, downhill locomotion is poorly studied [10]. There is also a toppling moment associated with downhill locomotion, but, unlike uphill locomotion, it causes a forward toppling moment that results in an increased reliance on braking. Most animals reduce their speed on declines, compared to level terrain locomotion [18, 20, 25, 26], but variables such as stride frequency exhibit large differences between studies [23, 24, 27]. There is a continuing lack of agreement in many parameters such as speed, muscle activity and the metabolic cost of locomotion on downhill surfaces [23, 27–30], along with the numerous different animal models that have been used, so currently it is difficult to determine if a common locomotor strategy exists for downhill surfaces (see Birn-Jeffery & Higham [10] for review). The specialised adhesive system on the feet of geckos likely further drives greater variation in strategies for moving downhill.

Geckos are a highly successful group, found in many ecological niches [31]. The adhesive system of geckos is an innovation that permits the occupation of ecological niches that were not previously available, thus allowing them to extensively radiate [32]. Gecko adhesion is very strong [33, 34] and achieved through close contact between the surface and setae on the ventral surface of the toes [35–37]. This close contact results in van der Waal interactions [38], frictional forces [39] and contact electrification [40], which collectively result in adhesion. To prevent adhesive forces on surfaces that do not require adhesion, such as level terrain, geckos keep their digits in a hyperextended position [21]. This prevents the setae from contacting the surface and thus prevents interactions such as van der Waals. Previous work has focussed on these adhesive mechanisms, including the physical interactions causing adhesion [33, 38–41] and the stereotyped micromechanics of the setae [42–47]. Yet, we currently do not understand how the adhesive system constrains overall body and limb mechanics, which is likely, given the requirement of stereotyped motions to engage and disengage this innovation.

The impact of the adhesive system on locomotor kinematics among geckos is poorly understood (but see [48]); but spatiotemporal characteristics have previously been studied. The gaits that geckos use on level terrain are similar to other legged animals (i.e. vaulting and bouncing gaits [49]). Rather than changing speed through a combination of stride length and stride frequency, geckos commonly rely solely on alterations in stride frequency on both level and inclined terrains [22, 50–52]. The adhesive system may play a key role in successful incline locomotion, but it could also severely constrain locomotion. This is evident in the lack of change in timing of deploying and detaching the adhesive system between different terrains [21, 52, 53], and reductions in maximal speed associated with larger toe pad areas [54]. Therefore we still do not understand the impact that the adhesive system has on locomotion.

Using a generalist pad-bearing gecko, *Chondrodactylus bibronii*, we investigated whole body and limb kinematics on differing slopes (both uphill and downhill). Although *C. bibronii* is considered a generalist [55], they are highly capable climbers with a good adhesive system [56]. Due to the specialisation of the manus and pes for accommodating the adhesive system, we expect that distal limb movements will be less modulated and more stereotyped to provide successful adhesion and detachment. We therefore hypothesise that geckos will modulate humeral/femoral and elbow/knee motions to a greater extent than more distal limb segments, with this effect becoming emphasised on steeper slopes in order to overcome gravity. We expect that proximal forelimb joints will undergo smaller angular excursions on steep declines to prevent toppling downhill, whilst on steep inclines greater angular excursions will provide increased contact time over which to produce extra work to climb uphill. As with previous incline studies we expect that speed will be reduced on inclines, but that this will be adjusted mainly by changes in stride frequency.

## Results

### Spatio-temporal characteristics

Neither 10° incline nor decline conditions had any significant differences in spatio-temporal variables (e.g. stance time, step length) in either forelimbs or hind limbs from level (Table 1). Forward speed and duty factor also did not significantly differ between any conditions (Table 1). Thus due to the low range of speeds observed the effects of speed on both limb movements and spatio-temporal variables are negligible. Swing time was significantly reduced by 50 % and 17 % in the forelimb and hind limb respectively, for the -45° condition compared to all other slopes (Table 1). The reduction in swing time only resulted in a significant decrease in stride time in the forelimb (Table 1). Stride frequency significantly increased by 54 % in the forelimb as compared to level, but was not significantly different to the level stride frequency in the hind limb (Table 1). Step and stride length were significantly reduced by 26 % on the -45° slope in the hind limb as compared to all other conditions (Table 1).

**Table 1:** Means and ANOVA results for spatio-temporal variables for the forelimb and hind limb

	Mean ± s.e.m.	Difference from level				F-statistic (degrees of freedom)	*P*-value
	Level	+10°	+45°	-45°	-10°		
Velocity (ms^-1^)	0.57 ± 0.067	0.03 ± 0.061	-0.02 ± 0.052	0.12 ± 0.057	-0.05 ± 0.031	-	-
Velocity (ms^-1^/sqrt(ms^-2a^SVL))	0.67 ± 0.055	0.02 ± 0.059	-0.01 ± 0.053	0.13 ± 0.065	-0.06 ± 0.029	1.50 (4,20)	0.24
Forelimb							
T_stance_ (s)	0.08 ± 0.008	-0.01 ± 0.008	0.00 ± 0.010	-0.02 ± 0.008	0.01 ± 0.007	1.54 (4,20)	0.23
T_swing_ (s)	0.06 ± 0.003	0.00 ± 0.002	0.00 ± 0.004	-0.03 ± 0.003^a^	0.00 ± 0.004	9.61 (4,20)	<0.005
T_stride_ (s)	0.14 ± 0.010	-0.01 ± 0.010	0.00 ± 0.011	-0.05 ± 0.010^a^	0.01 ± 0.009	3.61 (4,20)	0.02
DF	0.56 ± 0.024	-0.02 ± 0.020	0.00 ± 0.030	0.07 ± 0.025	0.01 ± 0.016	1.13 (4,20)	0.37
Stride Freq (Hz)	7.76 ± 0.592	0.07 ± 0.56	0.03 ± 0.588	4.16 ± 0.977^a^	-0.42 ± 0.415	5.45 (4,20)	<0.005
Step Length (m/SVL)	0.58 ± 0.009	-0.01 ± 0.007	-0.05 ± 0.009	-0.10 ± 0.002	-0.00 ± 0.007	1.13 (4,20)	0.31
Stride Length (m/SVL)	0.91 ± 0.015	0.00 ± 0.022	-0.08 ± 0.009	-0.17 ± 0.014	0.14 ± 0.047	0.76 (4,20)	0.40
Hind limb							
T_stance_ (s)	0.09 ± 0.008	-0.00 ± 0.014	0.02 ± 0.013	-0.01 ± 0.015	0.02 ± 0.007	1.30 (4,20)	0.30
T_swing_ (s)	0.05 ± 0.003	0.00 ± 0.002	-0.00 ± 0.003	-0.01 ± 0.003^a^	0.00 ± 0.003	4.56 (4,20)	0.008
T_stride_ (s)	0.14 ± 0.009	-0.00 ± 0.015	0.02 ± 0.015	-0.03 ± 0.018	0.02 ± 0.006	1.85 (4,20)	0.16
DF	0.62 ± 0.022	-0.01 ± 0.038	0.04 ± 0.021	0.02 ± 0.033	0.04 ± 0.025	0.48 (4,20)	0.75
Stride Freq (Hz)	7.51 ± 0.521	-0.17 ± 0.797	-0.44 ± 0.708	2.36 ± 1.264	-1.18 ± 0.358	2.75 (4,20)	0.06
Step Length (m/SVL)	0.61 ± 0.018	-0.03 ± 0.024	-0.05 ± 0.009	-0.16 ± 0.023^a^	-0.05 ± 0.009	8.21 (4,20)	<0.005
Stride Length (m/SVL)	0.99 ± 0.046	-0.06 ± 0.053	-0.10 ± 0.035	-0.25 ± 0.020^a^	-0.12 ± 0.041	4.36 (4,20)	0.02

*T* is time, *DF* is duty factor and *SVL* is snout-vent length

^a^Denotes significant differences from level

### Posture changes on sloped terrain

Shoulder and hip height at footfall (FF), mid-stance (MS) and end stance (ES) on inclines did not significantly differ from level (Table 2). The virtual leg length at FF increased in the hind limb on the downhill 45° condition (level: 0.021 m; -45°: 0.024 m), but significantly decreased at ES (level: 0.042 m; -45°: 0.039 m; Table 2). The elevation angle (angle of the virtual leg from the ground along the vertical-medio-lateral plane) was significantly reduced in the forelimb at ES on -45° incline as compared to level (Table 2). Hind limb elevation angle was significantly reduced at FF, from level, in the -45° condition (Table 2). The placement of the forelimb foot (azimuth angle) did not significantly change from level in any of the conditions (Table 2), but the hind limb at FF, in the -45° condition was placed in a significantly more posterior position compared to all other conditions (level: 171.49°; -45°: 201.82; Table 2).

**Table 2:** Means and ANOVA results for postural variables for the forelimbs and hind limb

	Mean ± s.e.m.	Difference from level				F-statistic (degrees of freedom)	*P*-value
	Level	+10°	+45°	-45°	-10°		
Forelimb							
L_FF_ (m/SVL)	0.60 ± 0.016	0.02 ± 0.004	0.01 ± 0.007	-0.00 ± 0.011	0.01 ± 0.004	0.00 (1,7)	0.98
L_ES_ (m/SVL)	0.28 ± 0.014	0.00 ± 0.008	-0.02 ± 0.008	0.01 ± 0.009	0.01 ± 0.010	1.78 (4,20)	0.18
L_MS_ (m/SVL)	0.39 ± 0.014	-0.00 ± 0.004	-0.00 ± 0.006	0.03 ± 0.009	0.00 ± 0.008	0.13 (2,18)	0.88
Shoulder height FF (m/SVL)	0.18 ± 0.010	-0.01 ± 0.009	-0.01 ± 0.012	-0.03 ± 0.010	0.00 ± 0.009	1.27 (4,20)	0.32
Shoulder height MS (m/SVL)	0.17 ± 0.009	-0.01 ± 0.004	-0.00 ± 0.009	-0.03 ± 0.009	-0.01 ± 0.008	2.34 (4,20)	0.09
Shoulder height ES (m/SVL)	0.17 ± 0.009	-0.01 ± 0.007	-0.01 ± 0.010	-0.02 ± 0.009	-0.01 ± 0.010	0.46 (4,20)	0.77
L_Azimuth_ FF	107.11 ± 1.094	-4.29 ± 0.919	1.46 ± 0.841	-5.43 ± 0.690	-4.80 ± 1.419	0.65 (1,7)	0.44
L_Azimuth_ ES	160.41 ± 3.402	3.85 ± 3.228	2.31 ± 2.725	-12.33 ± 4.711	-3.83 ± 3.060	1.88 (4,20)	0.15
L_Elevation_ FF	20.14 ± 0.222	-0.66 ± 0.666	0.31 ± 0.827	-5.31 ± 0.095	-0.68 ± 0.411	0.45 (1,7)	0.52
L_Elevation_ ES	52.19 ± 2.060	0.05 ± 1.884	-7.17 ± 2.281	-17.61 ± 2.079^a^	-2.88 ± 3.204	8.77 (4,20)	<0.005
Hind limb							
L_FF_ (m/SVL)	0.29 ± 0.009	0.01 ± 0.007	-0.01 ± 0.006	0.04 ± 0.019	0.03 ± 0.010	3.11 (3,15)	0.06
L_ES_ (m/SVL)	0.57 ± 0.013	0.02 ± 0.009	0.02 ± 0.006	-0.04 ± 0.012^a^	0.00 ± 0.011	4.76 (4,20)	0.007
L_MS_ (m/SVL)	0.39 ± 0.010	0.02 ± 0.012	0.02 ± 0.005	0.03 ± 0.011^a^	0.03 ± 0.010	3.22 (4,20)	0.03
Hip height FF (m/SVL)	0.19 ± 0.010	0.00 ± 0.015	-0.01 ± 0.012	-0.04 ± 0.017	-0.03 ± 0.010	1.03 (4,20)	0.42
Hip height MS (m/SVL)	0.17 ± 0.007	0.00 ± 0.013	-0.02 ± 0.009	-0.03 ± 0.013	-0.01 ± 0.008	0.94 (4,20)	0.46
Hip height ES (m/SVL)	0.18 ± 0.007	0.01 ± 0.011	-0.01 ± 0.007	-0.03 ± 0.010	-0.02 ± 0.005	0.99 (4,20)	0.43
L_Azimuth_ FF	171.49 ± 1.943	12.27 ± 5.961	12.89 ± 3.296	30.33 ± 6.793^a^	4.08 ± 2.203	4.22 (3,15)	0.02
L_Azimuth_ ES	248.55 ± 2.038	-1.62 ± 1.838	-1.44 ± 2.178	-3.33 ± 4.073	-7.09 ± 1.918	0.78 (4,20)	0.55
L_Elevation_ FF	45.01 ± 1.436	-0.95 ± 1.200	-7.84 ± 1.164^a^	-14.50 ± 1.452^a^	-9.98 ± 2.514	9.49 (3,15)	<0.005
L_Elevation_ ES	19.22 ± 0.719	-1.06 ± 1.008	-3.98 ± 0.964	-3.22 ± 0.964	-3.03 ± 0.567	2.15 (4,20)	0.11

*L* is leg, *FF* denotes footfall, *ES* is end stance and *MS* is mid stance

^a^Denotes significant differences from level

### Limb kinematics

**Fig. 1:**
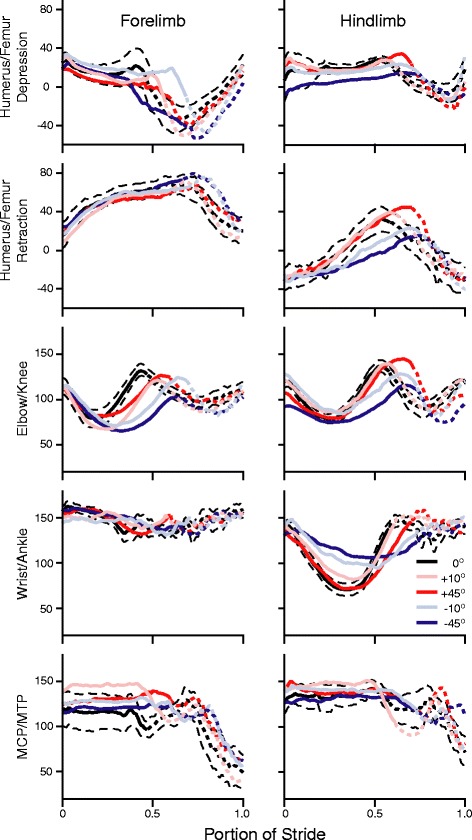
Average limb angle trajectory plots across all conditions for both forelimbs and hind limbs. *Solid lines* represent the stance phase, whilst *short dashed lines* represent the swing phase. The *long dashed*, *black lines* are the 95 % confidence intervals for the level terrain. The hind limb had the greatest limb angle trajectory changes from level condition compared to the forelimb, particularly at the ankle. The forelimb had greater timing changes associated with sloped locomotion

The forelimb joint angles, compared to the hind limb, had fewer significant changes on inclines compared to level (Fig. 1). The only significant changes in the forelimb, compared to level, were at ES; the elbow was significantly more flexed, by 25 %, on the -45° incline (*F*_4, 20_ = 4.19; *p* = 0.013) and the metacarpalphalangeal (MCP) joint was significantly more extended, by 33 %, on the 45° incline (*F*_4, 20_ = 3.91; *p* < 0.017). No significant differences from level occurred in humeral or pectoral girdle rotation in any of the other conditions.

**Fig. 2:**
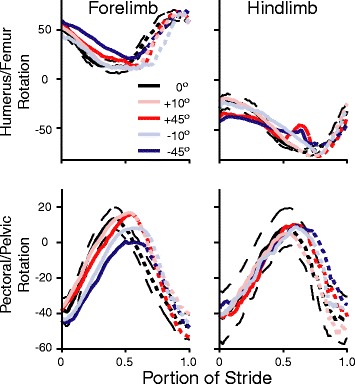
Average limb rotation and girdle trajectory plots across all conditions for both the forelimbs and hind limb. *Solid lines* represent the stance phase, whilst short dashed lines represent the swing phase. The long dashed, black lines are the 95 % confidence intervals for the level terrain. Girdle rotations are mostly symmetrical between the pectoral and pelvic girdles, suggesting one compensated for the rotation of the other. Femoral rotation changed the most at the two most extreme slope conditions

The hind limb joint trajectories deviated more from those on the level terrain in both the +45° and -45° conditions compared to the forelimbs (Fig. 1). Clockwise femur rotation was significantly greater at FF and MS on the -45° condition (Fig. 2; at MS: *F*_4, 20_ = 4.72; *p* = 0.007), and the femur was more depressed compared to level during MS and ES in that terrain. Incline did not significantly impact femur retraction. The knee was 22 % more flexed at FF for the -45° incline (*F*_4, 20_ = 7.75; *p* < 0.005), but did not significantly differ from level at either MS or ES. The ankle only significantly differed from level at MS for the -10° and -45° inclines (*F*_4, 20_ = 7.34; *p* < 0.005); in both cases the ankle was more extended by 37 % and 50 % respectively. As with pectoral girdle, the pelvic girdle had a consistently similar rotation trajectory in each condition (Fig. 2).

**Fig. 3:**
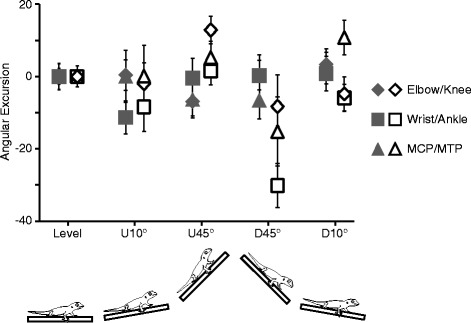
Average and s.e.m. boxplot of the change in stance angular excursion from the level condition. Grey symbols represent the forelimb whilst the open symbols represent the hind limb. Values above zero indicate increasing angular excursion whilst negative values indicate decreasing excursion. The hind limb significantly reduces the excursion of the more distal joints on the -45° condition resulting in the limb being maintained in a similar posture throughout stance at this slope

The angular excursion swept by each forelimb joint during the stance phase did not differ between any inclines. Conversely, there were significant reductions in the angular excursion of hind limb joints for the -45° incline compared to all other conditions (Fig. 3). The ankle (*F*_4, 20_ = 4.26; *p* = 0.012) swept through a significantly smaller range on the -45° condition as compared to all other inclines. The knee and metatarsalphalangeal (MTP) joints also swept through a smaller range of angles on the -45° condition by 19 % and 25 % respectively compared to all other conditions.

### Changes in modulation between limbs

In the majority of cases the hind limbs exhibited significantly greater modulation on inclined conditions compared to level terrain in contrast to the forelimbs (Table 3). In all cases where variables differed from level terrain at FF, such as humerus/femur rotation, depression and elbow/knee angle, the hind limb exhibited the greatest change in kinematics (Table 3). These differences were greatest on either the +45° or -45° inclines. Interestingly, the forelimb was modulated significantly more than the hind limb in humeral versus femoral rotation and depression at ES (Table 3).

**Table 3:** Significant t-test results for the modulation difference between fore and hind limbs

	Terrain condition	Forelimb difference from level	Hindlimb difference from level	T-statistic	*p*-value
FF humerus vs femur rotation	+45°	2.536	-14.993	2.205	0.043^a^
FF humerus vs femur rotation	-45°	9.902	-18.477	2.091	0.047^a^
ES humerus vs femur rotation	-45°	17.220	1.953	2.593	0.016
FF humerus vs femur depression	+10°	-0.571	15.229	3.413	0.006^a^
ES humerus vs femur depression	+10°	-29.867	-0.251	2.698	0.018
ES humerus vs femur depression	+45°	-14.100	0.593	3.22	0.006
ES humerus vs femur depression	-45°	-50.658	-11.626	3.93	0.001
FF elbow vs knee angle	-45°	-8.652	-27.044	2.152	0.047^a^
FF pectoral vs pelvic girdle rotation	-10°	-1.437	4.450	2.431	0.045^a^
FF pectoral vs pelvic girdle rotation	-45°	-2.497	14.812	2.499	0.032^a^
Angular excursion wrist vs ankle	-45°	0.213	-30.167	2.211	0.036^a^
Angular excursion MCP vs MTP	-45°	-6.531	-15.211	2.608	0.015^a^
FF Limb elevation angle	-45°	-5.307	-17.607	2.338	0.044^a^

Values are reported in degree (°) differences

*FF* is footfall and *ES* denotes end stance

^a^Represents where hind limbs were modulated to a greater extent than forelimbs

## Discussion

*Chondrodactylus bibronii* does not substantially change limb kinematics when moving on shallow slopes compared to level (Fig. 1). In addition, the geckos employed strategies to maintain speed on steep slopes (Table 1) via significant modulation of the hind limb whilst using similar forelimb movement patterns to those observed during level locomotion. Minimal changes in forelimb joint trajectories allow geckos to retain this limb to power or brake, depending on terrain. The hind limbs, on the other hand, are drastically adjusted on downhill slopes, resulting in them facing backwards, and it is therefore unlikely that they play any role in powering locomotion in these conditions [11]. Instead, the hind limb could be used to compensate for the braking requirements, any medio-lateral imbalance as well as dealing with the forward toppling moment during downhill locomotion. Where significant changes in limb kinematics occur for *C. bibronii*, they are generally limited to the more proximal joints. Therefore, although adhesion facilitates successful locomotion on many surfaces, it may also constrain locomotor mechanics. This may then result in large changes at the more proximal joints in non-level terrain, rather than adjusting kinematics at distal locations which may indicate a more feedforward driven locomotor control.

### Spatio-temporal characteristics

Geckos do not change the timing of limb movements on uphill surfaces compared to level (Table 1). This is drastically different to previous studies of inclined locomotion where significant increases in stride time [24, 57] and duty factor [13, 23, 24] are evident. Duty factor often increases on uphill surfaces, allowing greater time in contact with the ground to provide the extra work required to move against gravity. Collectively, this results in decreased speed on uphill surfaces [18, 19, 21, 58–61], but we do not observe this in the geckos in our study. Russell and Higham [21] examined species of pad-bearing and padless geckos, and found that a trade-off exists between adhesion on inclines and running speed. In this case, the pad-bearing species slowed down on inclines of 10° and greater compared to level locomotion, whereas the padless species did not [21]. In contrast, our results suggest that a trade-off may not exist for *C. bibronii* as they engage the adhesive system at steeper inclines without a decrease in running performance. The time to engage and disengage the gecko adhesive system does not change across conditions [52, 53], which suggests that *C. bibronii* is compensating for adhering to the surface by adjusting other parameters, such as stride frequency or length. Understanding the trade-off between using an adhesive system and performance in geckos requires future work to determine how and whether differing adhesive systems in geckos differentially affect locomotor performances on sloped surfaces.

One possible tactic for maintaining speed and stance time is to reduce swing time. Many previous studies have noted no significant changes in swing time on inclines compared to level [15, 28, 53, 62–67]. Remarkably swing time did decrease on the 45° decline condition from level in our study (Table 1). Reduction in swing time compared to level terrain has previously been noted in birds on inclines [23, 68]. Shortening the swing time, whilst keeping stance time constant, could be a strategy to maintain relatively consistent peak forces, as well as provide enough time for producing an impulse that can generate work for going uphill, or absorb energy (negative work) when moving downhill. Decreasing stance time, particularly in terrain conditions that require more work, also requires greater peak forces. Greater peak forces are associated with increased bone and muscle stresses [69], suggesting that animals may use strategies that allow the greatest time in contact with the ground to reduce these stresses. One strategy to do this, but still maintain speed, would be to reduce swing time as we observed for geckos.

A reduction in swing time also suggests a neuromechanical control strategy involving something other than passive pendular movements of the limb, where limbs swing purely under the effect of gravity [9, 70]. Instead, it is likely that the limb is swung faster, thus increasing the energetic cost of the swing phase [71, 72]. Several studies have noted swing leg velocity manipulations in non-level terrain [73–75], which can improve stability [76, 77] and can reduce peak leg forces and falls [78, 79]. The reduction in swing time in geckos will also bring the foot back into contact with the ground earlier, which is advantageous for an animal that has, and uses, an adhesive system. This reduction in swing time during downhill running may be a compensatory strategy to provide the animal with a greater portion of the stride in adhesion, but we still do not fully understand the consequences of shortening the swing time. As mentioned above, alteration to the leg swing can affect both the ability to successfully continue moving in complex terrain and leg forces, so future work should determine if a trade-off exists between increasing time for adhering and maintaining lower leg forces to reduce musculoskeletal stress.

### Posture changes on non-level terrain

Surprisingly, geckos do not crouch more on slopes as compared to level (Table 2). In our study, *C. bibronii*, maintained the level forelimb posture on all slopes, with only a slightly more crouched posture at ES on the -45° slope. The hind limb was more crouched on the +45° and -45° slopes, compared to level. Previous studies on a range of vertebrate taxa note a significantly more crouched posture on both uphill [13–16, 80] and downhill slopes [14–16, 30, 81], in a variety of gradients of 5-45° both on inclines and declines. Geckos are sprawled postured animals, and the species of gecko used here is small, so its centre of mass is closer to the substrate. Thus, if the sprawled posture is simply maintained then there might be a small and likely insignificant toppling moment associated with locomotion on slopes. This reduces the necessity for the gecko to change posture on slopes. Future work using force plates to allow measurements of centre of mass will corroborate this interpretation. Other small lizards do adopt a more crouched posture on inclines [13]; but these animals were run on dowels (curved surfaces), so a postural effect may be linked with perch shape rather than incline. Another aspect is the adhesive system; this species of gecko can adhere strongly to surfaces [56]. This may implicitly be an advantage, allowing them to adhere on both inclines and declines [11], thus removing the need for postural changes on slopes as the adhesive system is significantly overbuilt [33] and can provide enough adhesive force to counteract any toppling moments.

### Differential limb modulation

Hind limb kinematics on inclines and declines, relative to level, are modulated to a greater extent than forelimb kinematics (Table 3), suggesting a functional de-coupling of the two sets of limbs. Particularly in the -45° condition stance angular excursion in the more distal hind limb joints is greatly reduced than in other conditions (Fig. 3). Therefore, when moving on steep declines, *C. bibronii* significantly modifies the position of the hind limb, with digits becoming more posteriorly rotated [11]. Along with the reduction in angular excursion of the distal limbs, this results in the hind limb maintaining a constant rear-facing position throughout the stance phase. The hind limb is maintained in a more posterior position to allow the adhesive system to engage [11]. In particular, this rotation is partially achieved through the ankle, which exhibits a significantly lower angular excursion in the -45° condition, further suggesting a functional change in the hind limb. Conversely, the wrist joint trajectory remains constant across all conditions (Fig. 1). The maintenance of the wrist joint in geckos is very different to other lizard and mammal locomotor studies where the wrist undergoes significant changes dependent upon terrain condition [13, 57, 81, 82]. This suggests that the constraint seen in wrist movement in *C. bibronii* is likely a result of the adhesive system, but this does require future investigation in other geckos to determine if there is a link between wrist movement constraints and possession of an adhesive system.

Although the majority of changes occur in the hind limb, the movements of the forelimbs are also adjusted on the -45° condition. Humeral depression is reduced, resulting in greater humeral elevation at ES. When combined with a more flexed elbow (Fig. 1), this brings the front of the body closer to the surface, increasing the braking capacity of the limb but decreasing propulsion at ES. On steep downhill slopes the braking capacity is more important than propulsion as it helps prevent a headlong rush downhill. Locomotion on slopes appears to induce a de-coupling of limb function in animals where fore- and hind limbs are modified differently dependent on condition [19, 53, 83, 84]; on declines forelimbs are used for braking whereas on uphill slopes hind limbs power locomotion. Our current work indicates that geckos significantly modulate hind limb, but not forelimb, movements. The reduction of modulation in forelimbs, on both uphill and downhill conditions, may suggest that it is the dominant limb for providing work and power; this could be achieved on inclines by using the adhesive system to help pull the gecko upwards similar to chameleons [81, 85]. The hind limb also appears to have a greater range of motion [86], thus allowing it to be modulated to a greater extent than forelimbs on non-level terrain. This allows the hind limb to adjust to the locomotor requirements such as providing the necessary stability, both medio-laterally and cranio-caudally to prevent toppling in each terrain condition. However, future studies should obtain single forelimb and hind limb footfalls of geckos on force plates to define how the limbs function individually and in different terrain conditions.

*Chondrodactylus bibronii* minimally alters distal limb kinematics on sloped terrains compared to other animals. On 45° inclines the MCP is significantly more extended at ES whilst the MTP joint trajectory is steadily maintained on all conditions. This extension of the MCP may provide increased ‘push-off’ at ES with which to help propel the animal forward against gravity. Apart from this one difference, MCP and MTP joints are remarkably consistent on all other conditions, particularly during stance. The motion to engage and disengage the adhesive system is very stereotyped [36], and so geckos are more likely to alter proximal joints to provide extra work rather than altering distal joints that could affect the success of their adhesive capabilities [87]. Interestingly, this would be converse to the majority of other animals, where a proximal-distal gradient is observed. In these cases smaller feedforward adjustments in proximal joints provide the required work output dependent on condition, whilst distal elements can be rapidly altered via feedback to act as springs or dampers [28, 88–91]. Furthermore, kinematic studies on complex terrains show remarkable trends of increased changes, from level, in more distal elements rather than the proximal joints [14, 82, 92–94]. Therefore, how do geckos deal with perturbations if distal elements are restricted in their capacity for adjusting to terrain? Future work should further investigate how geckos adapt their locomotion to perturbations to provide insight of their neuromechanical control, whether as with other species a proximo-distal gradient occurs, or if the adhesive system has constrained and altered their neuromechanical control compared to other lizards. Although most work focuses on the benefits of adhesion, there might be a considerable cost associated with this innovation.

### Limb kinematics across lizard species

**Fig. 4:**
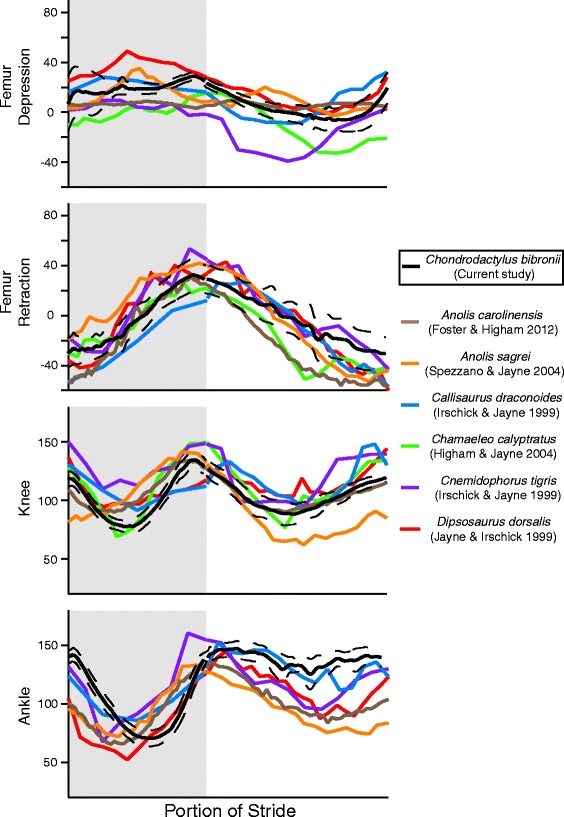
Comparison of hind limb angle trajectory plots across several lizard species. Data for species, apart from the current study’s *C. bibronii*, were obtained from previously published material as stated in the legend. The *light grey box* represents the stance phase. All data, separately for stance and swing, were interpolated to the same timings, so that speed was not a confounding factor and to observe the similarities and differences between trajectories easily. Femur retraction was remarkably similar across species, but the greatest differences occurred in the most distal element, the ankle. The gecko undergoes greater angular excursion during stance, whilst in the swing holding the ankle at a remarkably fixed angle compared to all other lizard species

We compare our gecko level terrain kinematics to those trajectories available in the literature as an initial analysis between a gecko and other lizards. This was only possible for the hind limb due to lack of data on forelimb kinematics for other lizards. Interestingly, femur retraction was consistent across all lizards with trajectories more or less overlapping during both stance and swing phases (Fig. 4). Conversely femur depression excursion was much smaller during stance in *C. bibronii* compared to all other lizards, particularly *Cnemidophorus tigris* (Fig. 4). Femur depression at ES in geckos is therefore unlikely to play an important role in the push-off phase of locomotion.

It is in the more distal limb elements where larger differences between gecko and other lizard kinematics were visible. Knee joint trajectory is similar across the arboreal species i.e. *C. bibronii, Anolis* and *Chamaeleo* [13, 94, 95], with all of these species exhibiting a large flexion-extension cycle during the stance phase. This is in stark contrast to the other more terrestrial, cursorial species that have a much smaller knee excursion (Fig. 4). This further suggests that ecology may drive the kinematics we observe, especially within a clade of animals.

However, the adhesive system of geckos also appears to play a role, with *C. bibronii* exhibiting greater ankle excursion during the stance phase than the other lizards (Fig. 4). *Chondrodactylus bibronii* also maintains the ankle at a very constant angle during the swing phase. Both the stance and swing attributes of *C. bibronii* locomotion suggest that the distal hind limb kinematics may be maintained across conditions to allow the stereotyped movement for engaging the adhesion system. Engaging the adhesive system on both inclines and declines is advantageous [11], as it prevents significant joint kinematic adjustments that would require changes in muscle gearing and neuromechanical control.

## Conclusions

*Chondrodactylus bibronii* uses significant de-coupling of the limbs to cope with steep downhill surfaces. The forelimbs undergo few kinematic changes on slopes compared to level terrain. Instead, geckos drastically alter hind limb kinematics, particularly on steep declines. It appears that, although the adhesive system is advantageous on inclines, a trade-off may exist between effective adhesion, and a dynamic response to perturbations, where previous studies have shown that distal limb elements compensate for changes in terrain. Further work should investigate the ability of geckos to cope with terrain perturbations. This innovation has significantly adjusted the biomechanics of gecko locomotion compared to other lizards, resulting in minimal adjustments in distal limb elements on non-level terrain in order to provide rapid and successful adhesion. Neuromuscular recordings would reveal if the recruitment of muscles has also been impacted. If there are changes in both mechanics and muscle activation, then there are likely major shifts in the evolution of neuromechanical control.

## Methods

### Animals

We used six adult *Chondrodactylus bibronii* (body mass = 12.83 ± 7.21 g [mean ± s.d.]; SVL = 73.3 ± 9.13 mm), obtained from local commercial suppliers. They were housed individually in 38 litre tanks. Each was fed *ad libitum* with crickets every other day, kept on a 12 h light/dark cycle and provided with a 100 W light. All experimental protocols and animal care were done under and approved by the University of California Riverside Institutional Animal Care and Use Committee following protocol number AUP A-20110038.

Each animal had markers placed on specific landmarks using white nail polish before running trials. Markers were placed on mid-pectoral/pelvic girdle and in-between the two girdles. Markers were further placed on the following joint centres: shoulder/hip, elbow/knee, wrist/ankle, and MCP/MTP (metacarpalphalangeal and metatarsalphalangeal) joints. These markers were only placed on the right side of the body, as only this side was filmed.

### Experimental protocol

Animals were run on a flat runway that was covered in 60-grit sandpaper and measured 0.96 m long and 0.14 m wide. The runway could be rotated to any angle and so the lizards were run on five different slopes: 0, +10, -10, +45, -45°. Three high speed video cameras (2 Phantom Miro M150, Vision Research Inc, New Jersey, USA; 1 Photron APX-RS, Photron, San Diego, CA, USA) were synchronously used to get one lateral and two oblique views of the lizards running. All cameras recorded at 1500 Hz, with the shutter speed set to 1/7000 s. Animals were encouraged to run across the runway by eliciting an escape response. We obtained at least 3 steady and straight strides for both forelimb and hind limb, condition and individual. We used only strides where the animal performed continuous movement and ran in a straight line. During post processing, we removed significantly unsteady strides in which speed changed more than 30 %. After data collection all videos were analysed using only every other frame. This allowed the retention of limb movement detail, particularly during the swing phase, but reduced the number of frames to digitise.

### Data analysis

All markers were digitised using DLT_dv5 [96], custom software for Matlab. Two extra points were also digitised; these were digit III tip on both the manus and the pes. The digitised landmarks provided three-dimensional coordinates for each marker, where the x, y, and z direction described the fore-aft, vertical and medio-lateral planes, respectively. All further analysis of the coordinates was processed using custom written code in Matlab (R2013b, The MathWorks, Natick, MA, USA).

A proxy for the centre of mass (CoM) was calculated from the average of three markers along the body mid-line. This proxy for the CoM was used to calculate overall animal speed and accelerations. Stride length and time were based from when a foot contacted the ground until the subsequent time that same foot contacted the ground. Stride length was only measured along the fore-aft axis. Duty factor was the time a foot was in contact with the ground divided by the stride time of that foot. Shoulder/hip height was calculated as the vertical distance between the ground and the shoulder/hip landmark.

**Fig. 5:**
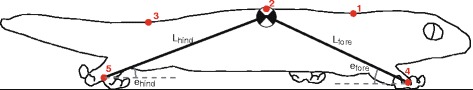
Schematic representation of measured variables. Markers 1-3 represent the body markers cranially, mid-way and caudually along the mid-line of the animal. Markers 4 and 5 represent the metacarpalphalangeal (MCP) and metatarsalphalangeal (MTP) joints respectively. *L* represents the virtual leg length between the centre of mass and the MCP and MTP. *e* represents the elevation angle along the medio-lateral and vertical axes, whilst the azimuth (not shown in the 2D figure) is the angle between the medio-lateral and fore-aft axes

A virtual leg was calculated as the 3-D distance between the centre of mass (CoM) and the metacarpalphalangeal (MCP) and metatarsalphalangeal (MTP) joints (Fig. 5). This leg measure represents a simplified point-mass model with a mass-less leg [9, 70], which describes locomotor gait across humans and non-human animals. Therefore calculating this variable during inclined locomotion allows us to observe how/if overall gait and posture are dependent on the slope. Associated with the leg length were two angles to describe the positioning of the limb. The first was the azimuth angle which describes the foot placement along the fore-aft-medio-lateral plane, measured from the left hand side of the medio-lateral axis. The second was the elevation angle measured along the vertical-medio-lateral plane which measures erectness of leg length (Fig. 5).

Finally, three-dimensional angles were calculated on all limb markers using previously published methods [13, 14, 94]. For elbow/knee, wrist/ankle, MCP/MTP angles, smaller values denote greater flexion at the joint. The shoulder/hip angles (movement of the humerus/femur) were represented by three different angles; depression, retraction and rotation. Each angle represents a two-dimensional component of the movement in the humerus/femur. Depression is the movement of the humerus/femur along the vertical plane. Angles less than 0° represent limb elevation and angles greater than 0° are limb depression. Retraction describes the movement of the humerus/femur along the fore-aft plane, where angles greater than 0° are protraction and less than 0° indicate limb retraction. Finally clockwise rotation of the humerus/femur are positive values whilst negative values are anti-clockwise rotation. Pectoral/pelvic rotation was also calculated. Values greater than 0° indicates that the left shoulder/hip is more anteriorly rotated than the right shoulder/hip. All angles were measured throughout time and at specific time points of footfall (FF), mid stance (MS) and at end stance (ES) events. Angular excursions were also calculated based on the minimum and maximum values during stance. All values provided in text are means ± s.e.m unless otherwise stated.

### Statistical analyses

All statistical analyses were performed using custom written codes in Matlab. Graphs were generated either in Matlab or Excel. Before performing any statistical analyses, and to remove the effect of individual from the data, a level average per individual was generated. This individual level average was then removed from each individual’s sloped conditions. All statistics were performed on the change from level data calculated per each individual. These were the data used to run ANOVAs, using slope as the main fixed factor and individual as the random factor. F-values were adjusted according to procedures set out by Zar [97]. Specifically, we use the mean squares of the interaction term (individual x slope) as the denominators when determining the F-values for the main slope effect. To directly assess the amount of limb modulation difference between fore- and hind limb, we ran T-tests. The data used for the t-tests were the absolute values of the values once the level mean was removed. Taking the absolute value allowed a comparison of magnitudes rather than direction changes from level.

## Abbreviations

CoM: centre of mass
DF: duty factor
ES: end stance
FF: footfall
Hz: hertz
L: virtual leg length measured from CoM position to base of foot
MCP: metacarpalphalangeal
MS: mid stance
MTP: metatarsalphalangeal
s.d.: standard deviation
s.e.m.: standard error of mean
SVL: snout-vent length
T: time
